# Cecal Gut Microbiota and Metabolites Might Contribute to the Severity of Acute Myocardial Ischemia by Impacting the Intestinal Permeability, Oxidative Stress, and Energy Metabolism

**DOI:** 10.3389/fmicb.2019.01745

**Published:** 2019-08-02

**Authors:** Lili Sun, Hongmei Jia, Jiaojiao Li, Meng Yu, Yong Yang, Dong Tian, Hongwu Zhang, Zhongmei Zou

**Affiliations:** Institute of Medicinal Plant Development, Chinese Academy of Medical Sciences, Peking Union Medical College, Beijing, China

**Keywords:** acute myocardial ischemia, gut microbiota, metabolomics, 16S rRNA gene sequencing, UPLC-Q-TOF/MS

## Abstract

Emerging evidence highlights the role of gut microbiota in regulating the pathogenesis of coronary heart disease. Here, we performed 16S rRNA gene sequencing and UPLC-Q-TOF/MS-based metabolomics to investigate the gut microbiome and metabolomes of cecal contents in the isoproterenol (ISO)-induced acute myocardial ischemia (AMI) rats. As expected, considerable gut microbiota alterations were observed in the AMI rats compared with the control rats, paralleling with intestinal inflammation and apoptosis. At phylum level, the abundance of *Firmicutes* was significantly decreased, whereas the abundance of *Bacteroidetes* and *Spirochaetae* was strikingly enriched in the AMI group. At genus level, the significant alteration of genera *Treponema 2*, *Rikenellaceae RC9 gut group*, *Prevotellaceae UCG-003*, and *Bacteroides* may contribute to the pathogenesis of AMI. These altered microbiota might influence the intestinal permeability and subsequently impair intestinal barrier and stimulate gut inflammation. Consistently, significantly metabolic differences of cecal contents between the AMI and control groups were revealed, and threonic acid, L-urobilin and L-urobilinogen were considered the most associated cecal metabolites with AMI. These strikingly altered metabolites were mainly related to energy metabolism and oxidative stress which could lead to apoptosis and further affect gut barrier. Ultimately, we revealed the potential link of these altered gut microbiota/metabolomes and intestinal inflammatory factors and apoptotic proteins and further confirmed their intimate connections with intestinal inflammation and gut barrier. Our findings depict uncovered potential relationship among the gut microbiome, cecal metabolomes and AMI.

## Introduction

Acute myocardial ischemia (AMI) refers to a pathological myocardial state, which occurs as a result of imbalance between myocardial oxygen demand and coronary blood supply (Kocak et al., 2016; Suchal et al., 2016). It is regarded as one of the leading causes of morbidity and mortality among humans worldwide (Nwokocha et al., 2017). Accumulating clinical evidence reveals that significant cardiovascular risk still exist even with the development of aggressive high potency medicine therapies and global cardiovascular risk reduction efforts (Lam et al., 2016). Recently, appreciation of the role of gut microbiota in human and animal health has exploded. The alteration of gut microbiota has been a focal point to interpret potential mechanisms of diseases. Emerging numerous studies highlight the role of gut microbiota in regulating the pathogenesis of coronary heart disease (Koeth et al., 2013; Tang et al., 2013; Gregory et al., 2015; Skagen et al., 2016; Zhu et al., 2016; Battson et al., 2018). As originally reported, there is a distinct association between the gut microbiota and the severity of MI in rats (Lam et al., 2012, 2016). A recent research has also revealed the changes of gut microbiota after ligating the left anterior descending artery in rats (Wu et al., 2017). These investigations underscore the potential connection of gut microbiota with AMI.

Meanwhile, there is a growing appreciation that gut microbiota was involved in host metabolic phenotypes. However, there is no available information with respect to intestinal metabolome following AMI, which related most to gut microbiota. Our previous study applied metabolomics to explore the metabolic functions in the development of disease. For instance, Yu et al. (2017) observed alterations of gut microbiota and fecal metabolic profiles in rats with depression. The results demonstrated that metabolomics make it possible to assess the variations of intestinal metabolic phenotype and decipher the pathological mechanism of AMI.

Isoproterenol (ISO) is a synthetic β-adrenergic agonist applied clinically to support heart rate and cardiac contractility (Zhou et al., 2017). However, excessive doses of ISO in rats were found to cause severe pathological changes in the myocardial tissue, giving rise to infarct-like necrosis of heart which is similar to MI observed in humans (Wei et al., 2013). So ISO is generally utilized for inducing MI in experiment animal models to obtain insight into the pathogenesis, diagnosis and efficiency therapy of AMI (Liu et al., 2014, 2016; Sahu et al., 2014). Cecal contents, with high density and diversity of bacterial, are suitable targets analyzing changes in intestinal microbial to mimic the microbial environment in humans (Zhang and Neau, 2002; Meng et al., 2014). Therefore, to deeply understand the relationship between gut microbial communities and AMI, we take advantage of the advances in high-throughput 16S rRNA gene sequencing and non-targeted metabolomics based on UPLC-Q-TOF/MS technique to reveal the alterations of gut microbial communities and characterize the metabolic phenotypes of cecal contents in ISO-induced AMI rats. We also elucidated the correlations between the gut microbiome/cecal metabolomes and inflammatory and apoptotic proteins to elucidate their potential functional interaction. This study may provide insights and basis for developing microbiota-based or intestinal metabolites-based prevention of AMI in the future.

## Experimental

### Reagents and Materials

HPLC-grade acetonitrile was purchased from J. T. Baker (Phillipsburg, NJ, United States). Ultrapure water (18.2 MΩ) was filtered using a Milli-Q water purification system (Millipore, MA, United States). Isoproterenol hydrochloride (ISO), formic acid, ammonium formate and leucine-enkephalin were bought from Sigma-Aldrich Inc. (St. Louis, MO, United States). The assay kits for aspartate transaminase (AST), creatine kinases (CK), creatine kinase-MB (CK-MB), lactate dehydrogenase (LDH), and superoxide dismutase (SOD) were obtained from Jiancheng Bioengineering Institute (Nanjing, China).

### Animal Treatments and Sample Collections

Healthy male Wistar rats (180 ± 20 g) obtained from the Institute of Laboratory Animal Science, CAMS and PUMC (Beijing, China), were housed in cages under the controlled environmental conditions of 20–25°C, 40–60% relative humidity, and a standard 12 h light/12 h dark cycle. The rats were fed with standard laboratory chow and purified water available *ad libitum* in Specific Pathogen Free Laboratory. After a week of acclimation, the rats were randomly allocated into two groups: control (*n* = 8) and AMI groups (*n* = 8). The model rats were treated with ISO as described in our previous studies (Liu et al., 2013, 2014, 2016). Briefly, the model rats were injected with ISO (85 mg/kg) by subcutaneous at an interval of 24 h at the last 2 days (on 27th and 28th day) in order to generate experimental AMI, while the control rats were received the same quantity of normal saline. After the last injection for 12 h, the electrocardiograms of normal and model rats were recorded continually after anesthetized with urethane by intraperitoneal injection (Queenthy and John, 2013). Then, the rats were sacrificed. The harvested plasma samples were centrifuged at 3000 rpm for 15 min at 4.0°C and were stored at −80°C until analysis. The lower portions of myocardial tissues for histopathology analysis were quickly collected and fixed in 10% buffered formalin solution for 48 h. Then, the cecal contents were collected and stored in sterile conical tubes, respectively, and were frozen at −80°C immediately for further microbial community and metabolomics analysis. At last, the ileum 5 cm above the cecum were cut off and were frozen at −80°C immediately for further western blot analysis. This study protocol was performed according to the guidelines and regulations for the Principles of Laboratory Animal Care and Use of Laboratory Animals published by NIH (NIH Publication, 8th Edition, 2011). All the experimental procedures were approved by the Ethics Committee of the Institute of Medicinal Plant Development, CAMS and PUMC.

### Histopathology

The myocardial tissues fixed in 10% buffered formalin solution were subjected to histopathological observations. The fixed myocardial tissues were embedded in paraffin, and were sectioned at 5 μm. Then the sections were stained with hematoxylin-eosin (H&E). The images were acquired by a light microscopy (Olympus, BX53, Japan).

### Biochemistry Assays

The biochemistry parameters of cardiac injury and oxidative stress were used to diagnose AMI, including AST, CK, CK-MB, LDH and SOD. These indexes of plasma were determined by a spectrophotometry (UV-3100, Mapada, China) using standard assay kits (Nanjing Jiancheng Institute of Biotechnology, Nanjing, China) according to the manufacturer’s protocol.

### 16S rRNA Microbial Community Analysis

The extractions of total DNA in cecal contents were performed using the E.Z.N.A.R Soil DNA Kits (Omega Bio-Tek, Norcross, GA, United States) as reported previously (Yu et al., 2017). The hypervariable regions V3-V4 of the 16S rRNA genes were chosen for pyrosequencing to investigate the taxonomic compositions of the microbial community. The PCR amplification was conducted in triplicate utilizing the barcoded universal bacterial primers 338F (5′-barcode-ACTCCTACGGGAGGCAGCA-3′) and 806R (5′-GGACTACHVGGGTWTCTAAT-3′). Triplicates were pooled, and the PCR amplicons were sequenced using an Illumina HiSeq platform. The raw sequence data were processed and analyzed with a QIIME software package. Then, sequences with a threshold of 97% similarity were assigned to the same operational taxonomic units (OTUs) on the basis of representative sequences using Usearch^1^ (Hartmann et al., 2012) and Greengenes Database^2^ (Desantis et al., 2006). R software (version 3.2.1) with the ‘vegan’ package was used to perform bacterial analysis of Bray-Curtis dissimilarities based on the levels of changed gut microbiota.

### Untargeted Metabolomics

#### Sample Preparations

The sample preparations were according to our previous published method with a little modification (Yu et al., 2017). Briefly, cecal contents were weighed on ice. 500 μL of cold water was added into cecal contents (100 mg). Then the sample tubes were vortexed (5 min) and centrifuged at 13 000 *g* for 15 min at 4°C. The suspension was transferred to a new 2-mL conical tube, and the residual pellet was further extracted using 500 μL of cold methanol. After vortexed (5 min) and centrifuged (13,000 *g* for 15 min at 4°C), both supernatants were combined and centrifuged at 13,000 *g* for 15 min at 4°C. The resulting supernatant was filtrated through a 0.22 μM membrane filter, and an aliquot of 3 μL was injected for the UPLC-MS analysis.

#### LC-MS Conditions

The UPLC-Q/TOF-MS analysis was carried out on a Waters Acquity^TM^ Ultra high Performance LC system (Waters Corp., Milford, MA, United States) combined with a BEH C18 column (2.1 × 100 mm, i.d. 1.7 μm, Waters Corp., Milford, MA, United States). The mobile phases were made up of (A) water-acetonitrile (95:5, v/v) and (B) acetonitrile-water (95:5, v/v), and each of the mobile phase including 2 mM ammonium formate and 0.1% formic acid. The elution gradient system for samples was as follows: 0–0.5 min, 0–1% B; 0.5–5 min, 1–30% B; 5–13 min, 30–50% B; and 13–17 min, 50–100% B. The flow rate was 0.45 mL/min. The auto-sampler compartment and column temperature were sustained at 4°C and 40°C, respectively.

The mass spectrometry (MS) was performed on a Waters SYNAPT G2 HDMS (Water Corp., Manchester, United Kingdom) TOF mass spectrometer equipped with an electrospray ionization source (ESI) in both positive and negative ion scan modes. The parameters were set as previously published (Yu et al., 2017): The capillary voltage was set at 3.0 KV (+) and 2.5 KV (−). The Sample and extraction cone voltage were set at 40 V and 4.0 V, respectively. The desolvation gas flow was set at 800 L/h with temperature of 400°C, and the cone gas flow was set at 40 L/h with source temperature of 100°C. Centroid data was collected from *m/z* 50 to *m/z* 1200 with a scan time of 0.15 s and an inter scan delay of 0.02 s, respectively. The lock mass in all samples was acquired by leucine-enkephalin [*m/z* 556.2771 (+) and *m/z* 554.2615 (−)] with a concentration of 0.5 μg/mL and a flow rate of 10 μL/min, to ensure the accuracy and reproducibility.

### Western Blot Analysis of Ileum Tissues

The ileum tissues were washed twice with cold PBS for the western blot analysis. The ileum sample was lysed in an appropriate volume of cold lysis buffer contained 1 mM PMSF. After incubated on ice for 20 min, the lysates were centrifuged at 13000 rpm for 20 min at 4°C. The level of total proteins was determined using the BCA assay kits according to the manufacturer’s protocol. The levels of TNF-α, IL-1β, cleaved caspase-3, and caspase-7 were measured as described in our previous study (Liu et al., 2016). GAPDH was used as the internal standard. The detail information of antibodies were summarized in Supplementary Table S1.

### Data Analysis

#### Statistical Analysis

The significance of differences between the groups was compared by the two-tailed Student’s *t*-test using the Statistical Package for Social Science program (SPSS 16.0, Chicago, IL, United States). The *p-*value < 0.05 was set as significance threshold for this study.

#### Multivariate Analysis

The raw MS data of samples were processed using MarkerLynx (Version 4.1, Waters Corp., United Kingdom). The obtained data set was then imported into SIMCA-P software package (v13.0, Umetrics, Umeå, Sweden) for principal component analysis (PCA) and orthogonal to partial least squares-discriminate analysis (OPLS-DA). PCA was employed to search for metabolic distinction and evaluate the predictive ability of the established model. OPLS-DA was applied to depict metabolic perturbation induced by AMI. In the OPLS-DA model, the variable importance in the projection (VIP) and *S*-plot statistics were used to select differential metabolites of ISO-induced AMI.

## Results

### Electrocardiogram Changes, Pathological Assessment, and Biochemistry Analysis of the ISO-Induced AMI

**FIGURE 1:**
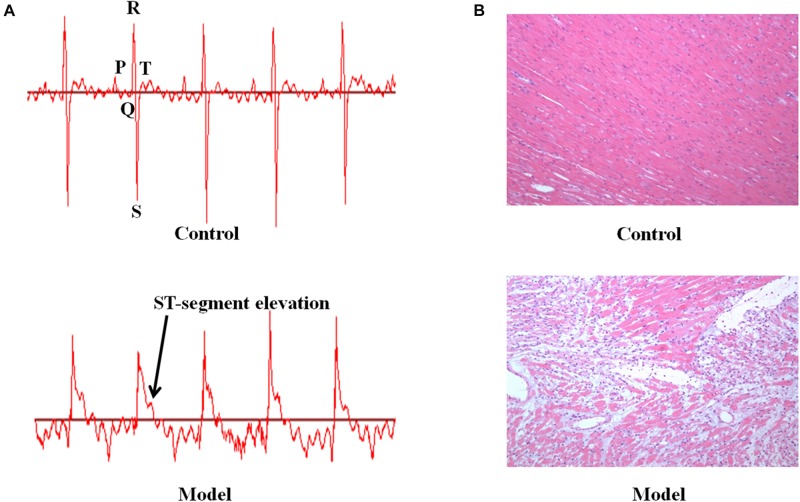
The ISO-induced AMI rats display ST-segment elevation and myocardium pathological change. **(A)** The representative electrocardiogram in the control and model groups. **(B)** The micrographs of myocardium for the experiment animals (HE, Original 100×).

We investigated the function of cardiac conduction system via electrocardiogram patterns in the control and AMI groups (Figure 1A). The AMI rats induced by ISO displayed significant elevation in ST-segment relative to the control rats. In addition, the morphological changes of myocardial tissues were described in Figure 1B. The myocardium of AMI group showed extensive structure disorders and classic pathological changes as compared with these presented in the control group, including watery degeneration, coagulation necrosis, neutrophilic granulocyte infiltration, capillary edema as well as scattered bleeding. Furthermore, AMI rats were biochemically diagnosed by determining plasma enzymes. Consistent with what was published in our previous study (Liu et al., 2013, 2014, 2016), the rats injected with ISO resulted in significant elevated levels of AST, CK, CK-MA, LDH and decreased level of SOD compared with the normal control (*p* < 0.001, Supplementary Figure S1). These results manifested that ISO-induced AMI model has been established successfully.

### Gut Microbiota Alterations in the ISO-Induced AMI

**FIGURE 2:**
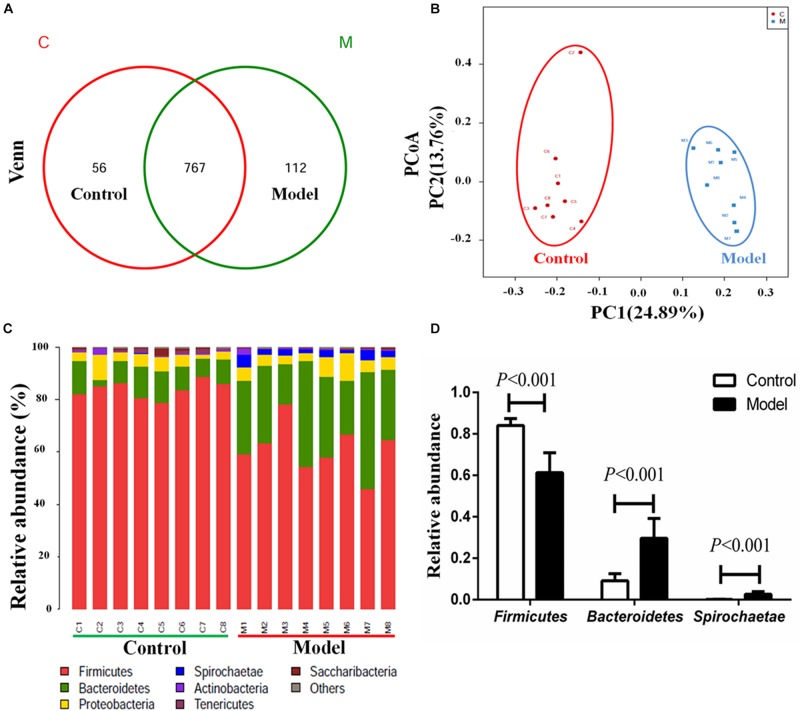
Isoproterenol-induced AMI leads to significant alterations of microbiota composition profiles in cecal contents. **(A)** Venn diagram of the shared OTUs between model group and control groups in cecal contents. **(B)** The PCoA score plots of cecal OTUs between the control and model groups. **(C)** The relative abundance of cecal microbiota at phylum level in the two different groups. **(D)** The relative abundance of *Firmicutes*, *Bacteroidetes* and *Spirochaetae* in the cecal control and model groups.

We applied 16S rRNA gene sequencing method to analysis the composition profiles of microbiota in the cecal contents. The Venn diagrams were used to display the shared OTUs. In the light of the Venn diagram of cecal contents, there were a total of 935 OTUs in the control and model groups, and 767 shared OUTs between the two groups, 112 unique OUTs (12.74%) in the model group, 56 unique OUTs (6.80%) in the control group (Figure 2A). Moreover, Principal coordinate analysis (PCoA) was employed to compare bacterial community patterns. The PCoA score plot based on sequences with a threshold of > 97% similarity at the OTU level revealed significant separation of the cecal community compositions between the model and control groups (Figure 2B), further corroborating the likely close relationship between AMI and microbiota. The PCoA score plot based on the analysis of weighted unifrac and unweighted unifrac were shown in the Supplementary Figure S2.

Concomitantly, the obviously separation of cecal community composition were further confirmed by hierarchical clustering analysis (Supplementary Figure S3). We then compared the bacterial compositions between the model and control rats at phylum and genus levels. As illustrated in Figure 2, a total of seven bacterial phyla were identified as known classified bacteria, while the rest was allocated into other unclassified bacteria. We observed a diminished abundance of *Firmicutes* and an increased abundance of *Bacteroidetes* and *Spirochaetae* in the model group (Figure 2D). Impressively, we found that the abundance of *Spirochaetae* was almost zero in the control group, while strikingly increased in the model group. A previous report showed that the relative abundance of phyla *Spirochaetes* was markedly increased at day 7 after ligating the left anterior descending artery (Wu et al., 2017). Therefore, the significant increases of phyla *Spirochaetae* may be related to the pathological process of AMI.

**FIGURE 3:**
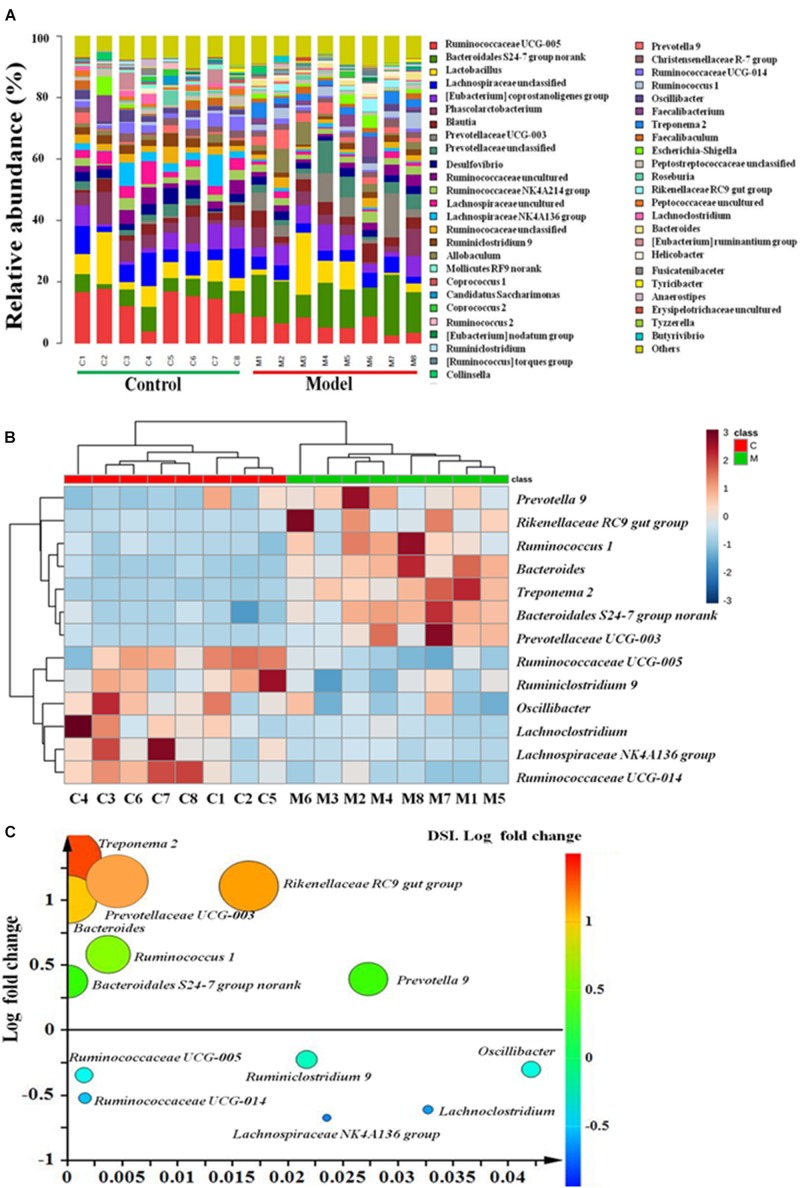
The gut microbiota at genus level in ISO-induced AMI occurs strikingly changes. **(A)** Relative abundance of gut microbiota at genus level in model and control groups. **(B)** The heat map summarizing the thirteen altered genera in ISO-induced AMI and control groups. Red, high concentrations; white, medium concentrations; blue, low concentrations. **(C)** Fold changes and *p*-values of significant altered genera in cecal contents in ISO-induced AMI compared with control group.

Furthermore, the relative abundance of cecal microbiota at the genus level also demonstrated a clear change in abundance among the two groups (Figure 3A). A total of thirteen genera displayed remarkable variations between the ISO-induced AMI and control groups. Among them, the abundance of seven genera (*Treponema 2*, *Rikenellaceae RC9 gut group*, *Prevotellaceae UCG-003*, *Bacteroides*, *Ruminococcus 1*, *Bacteroidales S24-7 group*, and *Prevotella 9*) were highly enriched, while other six genera (*Ruminococcaceae UCG-005*, *Ruminiclostridium 9*, *Oscillibacter*, *Lachno- clostridium*, *Lachnospiraceae NK4A136 group*, and *Ruminococcaceae UCG-014*) were considerably decreased in ISO-induced AMI group as compared with the normal control group (Figure 3B). Notably, the fold changes of *Bacteroides*, *Prevotellaceae UCG-003*, *Rikenellaceae RC9 gut group*, and *Treponema 2* were all larger than 10 (Figure 3C and Supplementary Table S2). We speculated that these four genera with high fold change maybe the most associated microbiota with AMI. And *Treponema 2* with the highest fold change (21.2741) belongs to the phylum *Spirochaetae* which has been reported to be related with myocardial infarction (Wu et al., 2017). Together, these results uncovered gut microbiota alterations in the ISO-induced AMI rats, indicating a condition of microbial dysbiosis.

#### Metabolic Profile Variations of ISO-Induced AMI

**FIGURE 4:**
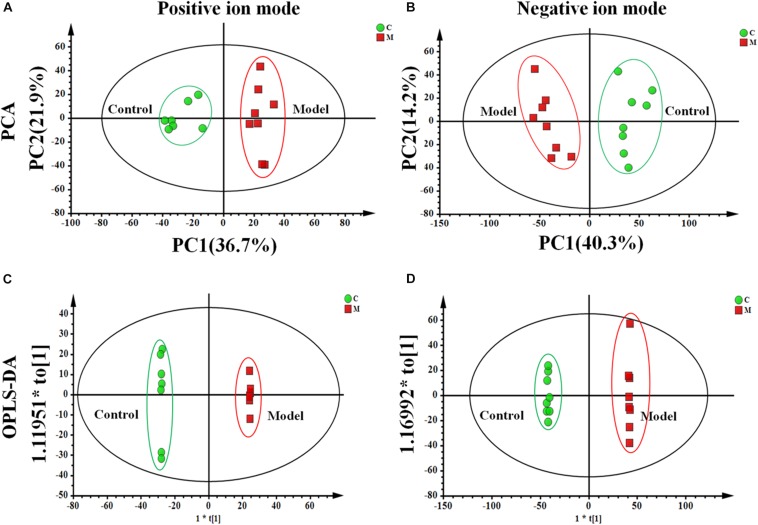
The metabolic profile of cecal contents in the ISO-induced AMI differs from that of in the control group. The PCA score plot of cecal contents between the control and model groups in **(A)** positive ion mode [*R*^2^*X* = 0.714, *Q*^2^ = 0.534] and **(B)** negative ion mode [*R*^2^*X* = 0.808, *Q*^2^ = 0.514]. The OPLS-DA score plots of cecal contents from the control and model groups in **(C)** positive ion mode [*R*^2^*X* = 0.803, *R*^2^*Y* = 0.998, *Q*^2^ = 0.923] and **(D)** negative ion mode [*R*^2^*X* = 0.806, *R*^2^*Y* = 0.999, *Q*^2^ = 0.955].

We used an untargeted metabolomics based on UPLC-Q-TOF/MS to depict metabolic profiling of cecal contents in the ISO-induced AMI rats and control rats. Supplementary Figure S4 showed the typical base peak intensity (BPI) chromatograms of cecal contents from each experiment group, respectively. The PCA analysis shown in Figures 4A,B exhibited distinct differences in metabolome between the control and model groups. OPLS-DA (Figures 4C,D) was performed to identify the metabolites that distinguish the control and model rats. Variables far from the origin in the *S*-plots (Supplementary Figure S5) with VIP values ≥ 1 and significant difference between the control and model groups (*p* < 0.05) was chosen as the differential metabolites responsible for the metabolic profile discrepancy induced by ISO. The metabolites were characterized by comparing the accurate mass, MS^*E*^ spectra obtained from UPLC-Q-TOF/MS with literatures and a variety of databases such as HMDB^3^, METLIN^4^, KEGG^5^, Chem- Spider^6^ and MASSBANK^7^.

Here, an example was used to elucidate the identification process of the differential metabolites. According to the MassLynx 4.1 software, the most likely ion elemental composition of **C7** (*m/z* 413.2670 at 11.28 min) was C_24_H_38_O_4_Na ([M+Na]^+^, calculated mass 413.2668). Candidates were gained by searching the HMDB, METLIN, and MASSBANK databases for further identification. As a results, only one compound (nutriacholic acid) was reported to be found in feces (MW tolerance to 0.01 Da), and the ion fragments *m/z* 355.2642 and *m/z* 309.2585 were consistent with the MS/MS spectra in the HMDB databases (Supplementary Figure S6). Therefore, the ion of *m/z* 413.2672 was tentatively identified as nutriacholic acid.

As illustrated in Supplementary Table S3 and Supplementary Figure S7, 13 variables were regarded as differential metabolites. The levels of threonic acid (**C1**), L-urobilinogen (**C6**), chenodeoxycholic acid (**C8**), and deoxycholic acid (**C12**) were markedly down-regulated, while the levels of tryptophanamide (**C2**), L-urobilin (**C4**), MG[18:2(9Z,12Z)/0:0/0:0] (**C5**), nutriacholic acid (**C7**), 4-(2-aminophenyl)-2,4- dioxobutanoic acid (**C9**), 2-hydroxyhexadecanoic acid (**C13**), and unidentified metabolites **C3**, **C10**, **C11** were significantly up-regulated in the ISO-induced AMI group compared with the control group. These metabolites were mainly involved in five metabolism pathways, including ascorbate and aldarate metabolism (**C1**), tryptophan metabolism (**C2**, **C9**), porphyrin and chlorophyll metabolism (**C4**, **C6**), fatty acid metabolism (**C5**, **C13**), and bile acid biosynthesis (**C7**, **C8**, **C12**).

Then, a comprehensive metabolic network was mapped by metabolic pathway analysis on MetaboAnalyst 3.0^8^. The importance of these pathways related to AMI was evaluated according to the impact value and –log(*p*). As evident in Supplementary Figure S8, ascorbate and aldarate metabolism (impact > 0.01) was regarded the most relevant pathway involved in AMI, and the next was porphyrin and chlorophyll metabolism (impact = 0.009). Taken together, these results revealed the significant alterations of cecal metabolic profile in ISO-induced AMI rats, and threonic acid (**C1**), L-urobilin (**C4**) and L-urobilinogen (**C6**) were likely the most important cecal metabolites in AMI rats.

### Expression of Inflammatory Factors and Apoptotic Proteins in Ileum

**FIGURE 5:**
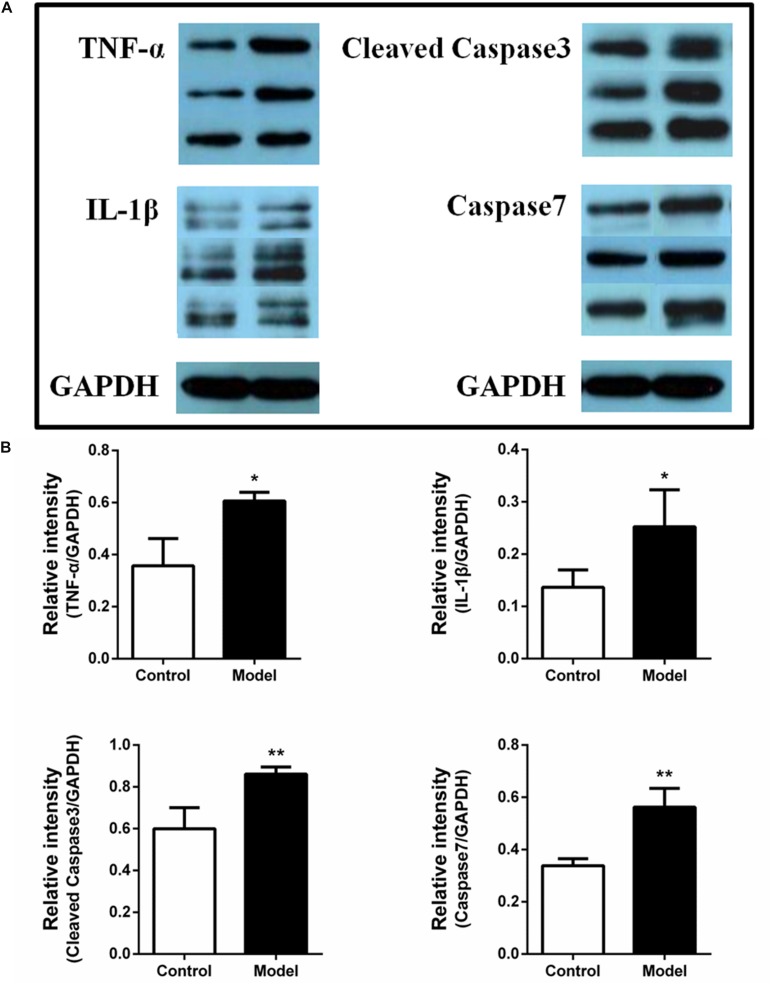
Isoproterenol-induced AMI rats are accompanied by the over-expression of inflammatory factors and apoptotic proteins in ileum. **(A)** The protein expression bands of TNF-α, IL-1β, cleaved caspase-3, and caspase-7 in ileum between the ISO-induced AMI group and model group. **(B)** The Bar graph of the over-expression of TNF-α, IL-1β, cleaved caspase-3, and caspase-7 in ileum between the ISO-induced AMI group and model group. ^*^p < 0.05 versus the control group, ^∗∗^*p* < 0.01 versus the control group.

A previous study revealed the occurrence of intestinal barrier impairment in AMI rats by ligating the left anterior descending artery (Wu et al., 2017). So it is possible that the AMI rats induced by ISO were paralleled with intestinal inflammation and apoptotic response, which may further impair the gut barrier. To further validate this hypothesis, we investigated the expression of TNF-α, IL-1β, cleaved caspase-3 and caspase-7 in ileum using western blot assay. The protein expression bands were displayed in Figure 5A. The results illustrated that the expression of TNF-α, IL-1β, cleaved caspase-3 and caspase-7 in ISO-induced AMI group were remarkably increased compared with the control group (Figure 5B, *p* < 0.01 or *p* < 0.05). The over-expression of inflammatory factors and apoptotic proteins in ileum may influence the function of intestinal, impair the intestinal barrier, and further affect the inflammation and apoptosis of whole cycle system and eventually the progression of AMI.

**FIGURE 6:**
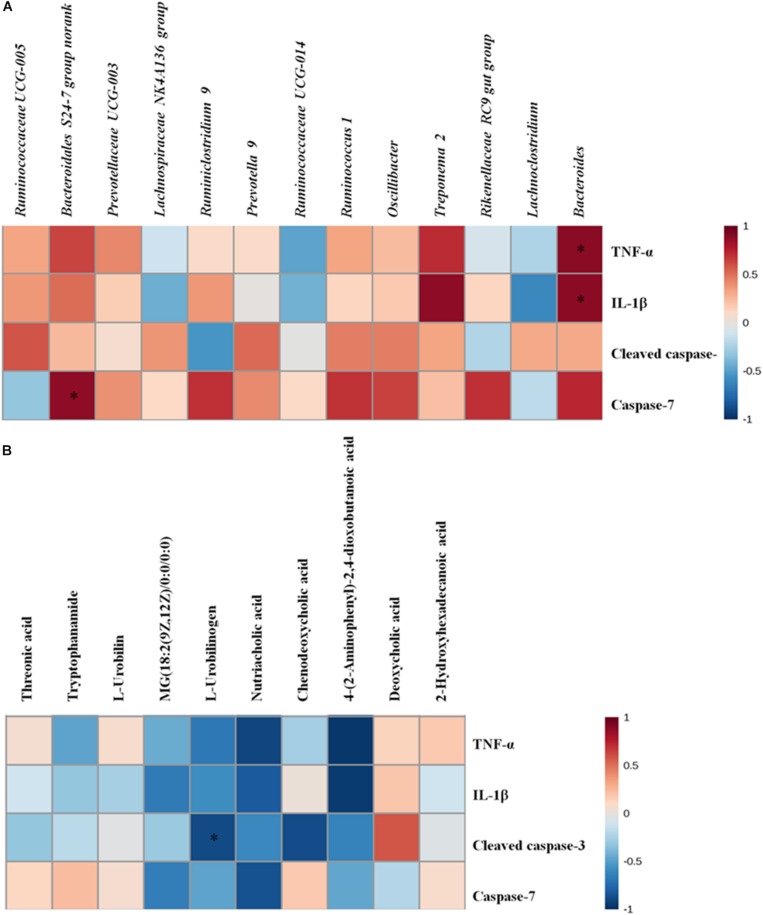
**(A)** The altered gut microbiota exhibit correlations with over-expressed inflammatory factors and apoptosis proteins. **(B)** The cecal biomarkers exhibit correlations with over-expressed inflammatory factors and apoptosis proteins. Correlation heat map of altered cecal microbiota genera/disturbed cecal metabolites and over-expressed inflammation factors and apoptosis proteins between the control and model groups; ^*^p < 0.05, ^∗∗^*p* < 0.01.

As we also observed significantly alterations of the gut microbiota and metabolites in cecal contents, we speculated that these alterations probably play a part in the development of intestinal inflammation and apoptosis. We performed data crosstalk among the gut microbiome/cecal metabolomes and over-expressed inflammatory factors and apoptotic proteins based on Pearson’s correlation coefficients in order to investigate their potential functional relationships. As displayed in Figure 6 and Supplementary Table S4, there were strong correlations between gut microbiota/cecal metabolomes and over-expressed inflammatory factors and apoptotic proteins. *Bacteroidales S24-7 group norank* was related positively with the level of caspase-7 (*r* = 0.88133, *p* < 0.05). Meanwhile, we also found strong positive relation between *Bacteroides* and the levels of two inflammation factors (TNF-α, *r* = 0.89094, *p* < 0.05; IL-1β, *r* = 0.88589, *p* < 0.05). Inversely, L-urobilinogen (**F9**) exhibited negative correlation with cleaved caspase-3 (*r* = −0.88540, *p* < 0.05). Taken together, the correlation analysis further suggested that the potential link tethering the cecal microbiome or metabolomes to AMI is conceivable and indicated that the gut microbiota or intestinal metabolism might exert effects on heart and systems either directly or indirectly.

## Discussion

Acute myocardial ischemia continues to be the main cause of death all over the world because the treatment of the majority of AMI patients remains unsatisfactory. Considering increasingly reports support the associations of gut microbiota and diseases, the regulation of gut microbiota may be developed to a therapeutic approach for common diseases. Previous studies have uncovered that gut microbiota serves an important function in coronary artery disease (Lam et al., 2012; Koeth et al., 2013; Tang et al., 2013; Gregory et al., 2015; Skagen et al., 2016; Zhu et al., 2016; Wu et al., 2017; Battson et al., 2018). They can affect the intestinal permeability, enhance the transfer of harmful substances in blood, and stimulate inflammatory response (Koutsos et al., 2015; Kelly et al., 2016). Thus, it’s highly warranted to characterize intestinal microbiota in AMI and identify microbial therapeutic targets. This motivated us to perform a comprehensive analysis of AMI on the gut microbiota.

Therefore, we performed 16S rRNA gene sequencing to investigate the effect of ISO-induced AMI on the gut microbiota. In our study, significant microbiota changes at both phylum and genus levels of cecal contents were observed in AMI rats induced by ISO. With regard to phylum level, the abundance of *Firmicutes* decreased and the abundance of *Bacteroidetes* and *Spirochaetae* enhanced in the AMI group compared to the control group. *Firmicutes* and *Bacteroidetes*, the two largest phyla inhabiting intestinal tract, are intimately associated with the healthy state of host. They are associated closely with the environment character and can be either helpful or problematic on human and animal health. Of note, *Bacteroidetes* were reported to be implicated in immune regulation including activation of inflammation and autoimmune diseases (Carr et al., 2002; Gibiino et al., 2018). Moreover, significant alteration of phyla *Firmicutes* and *Bacteroidetes* were also observed in coronary heart disease patients’ fecal samples. However, the change tendencies of them were in contrast to what we observed (Cui et al., 2017). One possible explanation for these results is that the different gut environments may affect the abundance and composition of gut microbiota. Wu et al. (2017) recently revealed that the gut microbiota was significantly altered at day 7 post AMI by ligating the left anterior descending artery, paralleled with impairment of gut barrier. They observed that the relative abundance of phyla *Synergistetes* and *Spirochaetes*, genus *Tissierella Soehngenia* were significantly increased at day 7 after AMI compared with SHAM group. So the remarkably increase of *Spirochaetes*, a kind of special bacteria with long spiral-shaped cells, might contribute to the pathogenesis of AMI. Thus, all these results indicated that the alterations of phyla *Firmicutes*, *Bacteroidetes*, and *Spirochaetes* might be involved in the incidence of cardiac events.

In addition, among the thirteen remarkably altered genera, *Bacteroides*, *Prevotellaceae UCG-003*, *Rikenellaceae RC9 gut group*, and *Treponema 2* with larger fold changes maybe the most related microbiota to AMI. *Bacteroides* is a kind of gram-negative anaerobia genus without spore, whereas *Prevotellaceae UCG-003* belongs to a relatively new bacterial family. Previous studies indicated that anaerobic organism such as *Bacteroides* can cause intra-abdominal infections (Brook, 2004), and the abnormal increase of *Prevotellaceae* abundance exacerbated the occurrence of inflammation (Elinav et al., 2011). In this study, the increases of *Bacteroides* and *Prevotellaceae-003* might relate to the severity of inflammatory in ISO-induced AMI. Besides, A previous study has found that the genus *Rikenellaceae RC9 gut group* was significantly increased in the high-fat diet with high-dose genistein mice group and may play a vital role in lipid metabolism (Zhou et al., 2018). In the present study, we also observed the significant enhancement of *Rikenellaceae RC9 gut group*. Thus, the increase of *Rikenellaceae RC9 gut group* might associate with the lipid metabolism. *Treponema*, belongs to the phylum *Spirochaetae*, was reported to be correlated positively with blood pressure (Marungruang et al., 2018) and its abundance was recently found to be associated with obesity in humans (Matsushita et al., 2015). This bacterium might also be implicated in periodontal disease which is a known risk factor for atherosclerosis (Marungruang et al., 2018). So the increase of *Treponema 2* in our study might be closely linked to AMI induced by ISO. It is well known that intestinal microbiota is a crucial factor in maintaining intestinal barrier and harmful metabolites’ transfer. The alterations of gut microbiota may destroy gut barrier integrity and promote transfer of harmful metabolites which induce inflammatory response (Di et al., 2016). Based on the above observation, we supposed that these altered genera might play a part of role in intestinal permeability and further affect intestinal barrier and gut inflammation. As far as we know, there is no any research reported that these genera are associated with AMI in humans or animal models. Further investigations are needed to explore the contributions of these bacteria to the gut barrier function, permeability and the development of AMI.

Recent numerous studies unraveled that metabolic alterations were paralleled with gut microbiota disorder during the development of diseases, such as atherosclerosis (Koeth et al., 2013), depression (Yu et al., 2017), obesity (Ley et al., 2006; Liu et al., 2017), hyperlipidemia (Le et al., 2013), IBS (Gall et al., 2011), and Crohn’s disease (Jansson et al., 2009). In our study, significant alterations of metabolites in cecal contents were also observed. Strikingly, ascorbate and aldarate metabolism (impact > 0.01) and porphyrin and chlorophyll metabolism were considered the most important pathways involved in AMI. Ascorbate and aldarate metabolism, belongs to carbohydrate metabolic pathways, can improve the resistance of body against diseases. Ascorbic acid (**C1**) is a degradation product of ascorbic acid (vitamin C) which exhibits a protective role against free radicals (Trezzi et al., 2017). Therefore, the decrease of **C1** in our study may interfere with ascorbate and aldarate metabolism and likely affect the oxidative stress response which is closely involved in the pathogenesis of AMI. L-urobilinogen (**C6**), an end-product of bilirubin, is transformed from L-Urobilin (**C4**). They are all participated in the Porphyrin and chlorophyll metabolism. Early research had described the protective effect of antioxidant bilirubin in liver disease (Chen et al., 2018). And the levels of **C4** and **C6** may reflect indirectly the level of bilirubin, which is related to the oxidative stress. The oxidative stress stimulates the generation of reactive oxygen species, leads to apoptosis which could further induce injury of gut barrier. Therefore, the changes of **C4** and **C6** in the present study might stimulate the oxidative stress response and apoptosis, and further affect the incurrence of AMI. In addition, among the five involved metabolic pathways, tryptophan metabolism and fatty acid metabolism anomalies have been also described in AMI animal models in previous metabolomics studies (Liu et al., 2013, 2014). They are both vital energy metabolisms. Thus, the aberrant increase of **C2, C5, C9**, and **C13** indicated that energy metabolism was disordered during the formation of ISO-induced AMI. Taken together, the significant changes of intestinal metabolism in AMI rats might couple with oxidative stress response and energy metabolisms aberrance.

Due to the potential association between these altered genera/metabolites and inflammation and gut barrier, we then determined the expression of inflammatory factors and apoptotic proteins in ileum. The over-expression of TNF-α, IL-1β, cleaved caspase-3 and caspase-7 in model ileum indicated that ISO-induced AMI may be companied by gut barrier damage which may be associated with the alterations of gut microbiome or metabolomes. In other word, these altered gut microbiome or metabolomes might further promote inflammatory and apoptotic response and contribute to the development of AMI. The results of correlation analysis further confirmed this hypothesis. So therapeutics targeted gut microbiota or metabolites could be developed in treating inflammatory and cardiac diseases.

It should be noted that these potential microbiome and metabolomes are limited to triggering AMI-related molecular mechanisms and are unlikely to be the sole player in AMI. The effects of gut microbiota and intestinal metabolites are most likely part of a much broader, multifactorial processes. Further studies are necessary to clarify and validate the function of the closely correlated genera or intestinal metabolites and to deep explore the interaction of gut microbiota and ISO-induced AMI. While acknowledging the possibility of the gut microbiota-host interplay, these gut microbiota and intestinal metabolites that didn’t show significant correlation with AMI are still valuable and warrant further studies.

## Conclusion

The pleiotropic effects of gut microbiota on host physiology have been well known. We demonstrated that ISO-induced AMI affected the compositions of gut microbiota and that this effect was accompanied by the alterations of cecal metabolic profiles and the intestinal inflammation and apoptosis. What’s more, the altered gut microbiota and cecal metabolomes showed highly interconnection with inflammatory factors or apoptotic proteins. Four genera (*Treponema 2*, *Rikenellaceae RC9 gut group*, *Prevotellaceae UCG-003*, and *Bacteroides*) and three cecal metabolites (threonic acid, L-urobilin, and L-urobilinogen) maybe relate most to the development of AMI. Their changes might affect the intestinal permeability, oxidative stress and energy metabolism, which might further stimulate the intestinal inflammation and gut barrier injury and contribute to the severity of AMI. Our observations further support the view that the molecular crosstalk between the gut microbiota and host is of utmost importance for health. Overall, our findings highlighted the need for integrative analyses of gut microbiota and metabolomes, and extended insights into the connections among the gut microbiome, metabolomes and AMI. Elucidating the mechanism by which gut microbiota or intestinal metabolites affect AMI could develop new prevention and diagnosis to promote AMI patients’ health.

## Data Availability

The raw data supporting the conclusions of this manuscript will be made available by the authors, without undue reservation, to any qualified researcher.

## Ethics Statement

This study was carried out in accordance with the recommendations of the Principles of Laboratory Animal Care and Use of Laboratory Animals published by NIH. The protocol was approved by the Ethics Committee of the Institute of Medicinal Plant Development, CAMS and PUMC.

## Author Contributions

LS, HJ, and ZZ conceived and designed the experiments. LS and ZZ were responsible for drafting the article. LS, HJ, JL, MY, YY, DT, and HZ were involved in the experiments preformation and data analysis. All authors approved the final version of the manuscript.

## Conflict of Interest Statement

The authors declare that the research was conducted in the absence of any commercial or financial relationships that could be construed as a potential conflict of interest.

## Footnotes

1 http://www.uio.no/english/services/it/research/hpc/abel/help/software/USEARCH.html

2 http://greengenes.lbl.gov/

3 http://greengenes.lbl.gov/

4 http://metlin.scripps.edu

5 http://www.genome.jp/kegg/

6 http://www.chemspider.com/

7 http://www.massbank.jp/

8 http://www.metaboanalyst.ca/
