# Supplementary material for: Cecal Gut Microbiota and Metabolites Might Contribute to the Severity of Acute Myocardial Ischemia by Impacting the Intestinal Permeability, Oxidative Stress, and Energy Metabolism
Source: Front Microbiol. 2019 Aug 2;10:1745. doi: 10.3389/fmicb.2019.01745 (PMC6687875; doi:10.3389/fmicb.2019.01745)
Supplement: Supplementary file 1 [file Data_Sheet_1.docx]

Supplementary Material

## Supplementary Figures


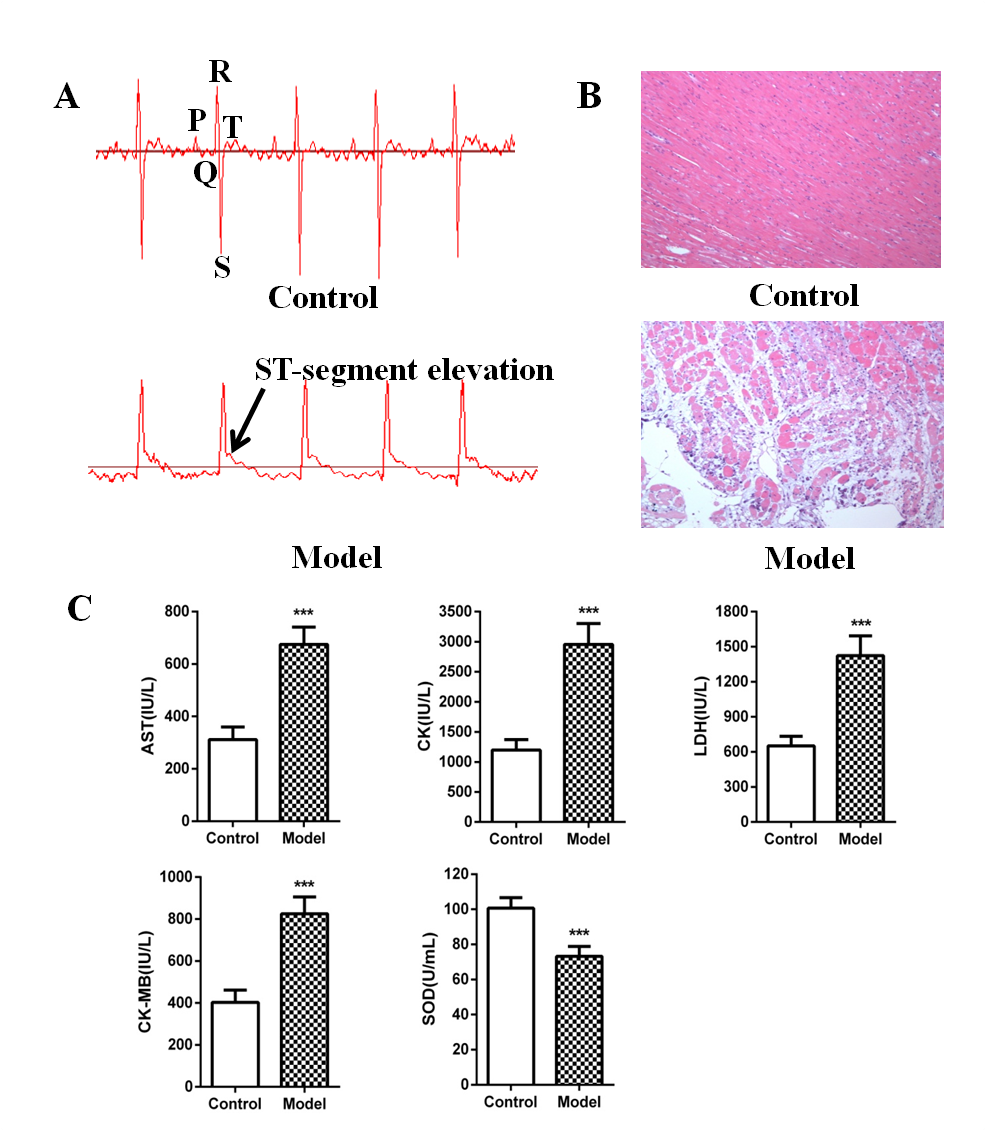


**Figure S1.** The plasma biochemical parameters of the two experimental groups. Values were expressed as mean ± SD (n = 6). ^***^*p* < 0.001 versus the control group.


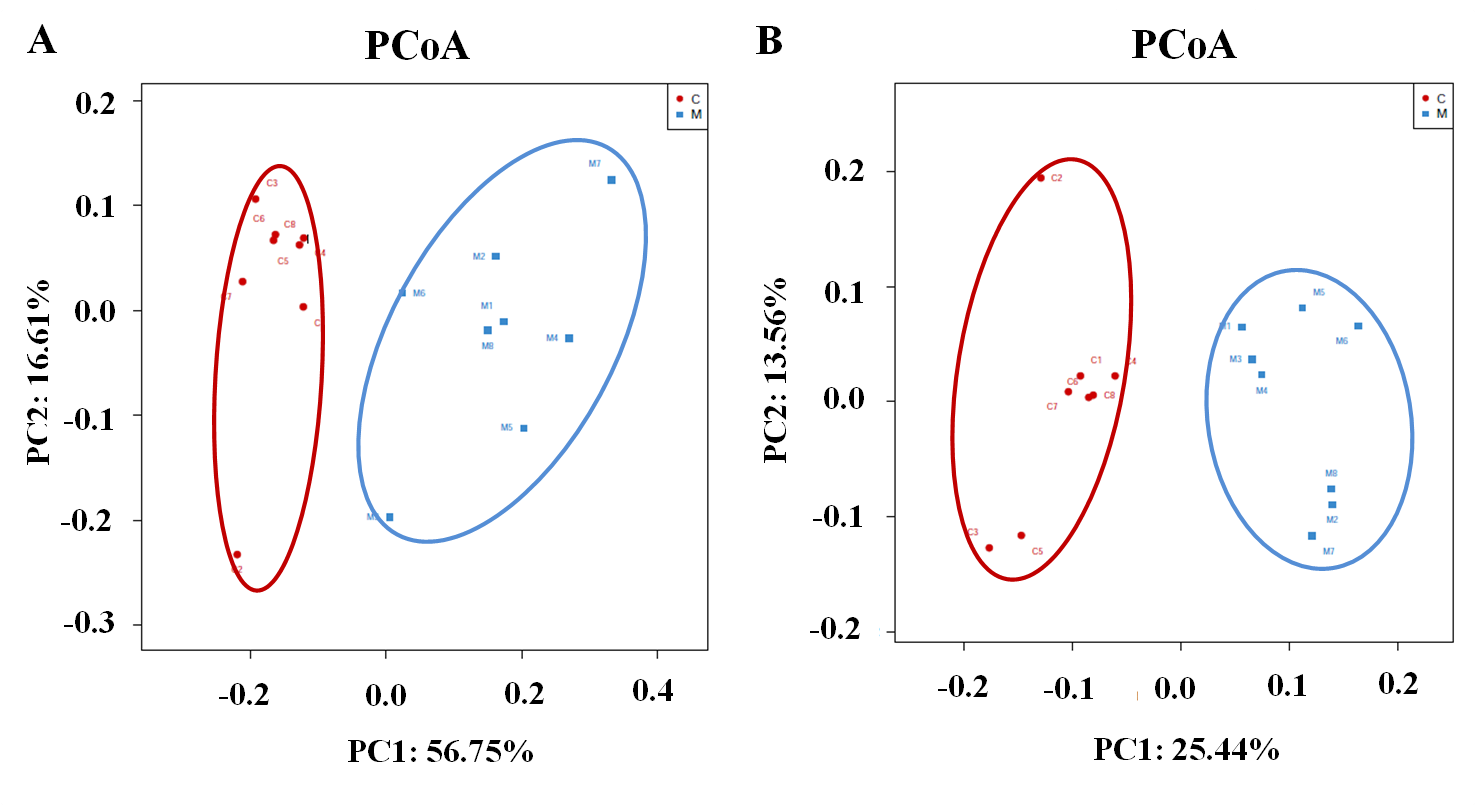


**Figure S2.** The PCoA score plots of cecal OTUs between the control and model groups based on the analysis of (A) weighted unifrac (B) unweighted unifrac


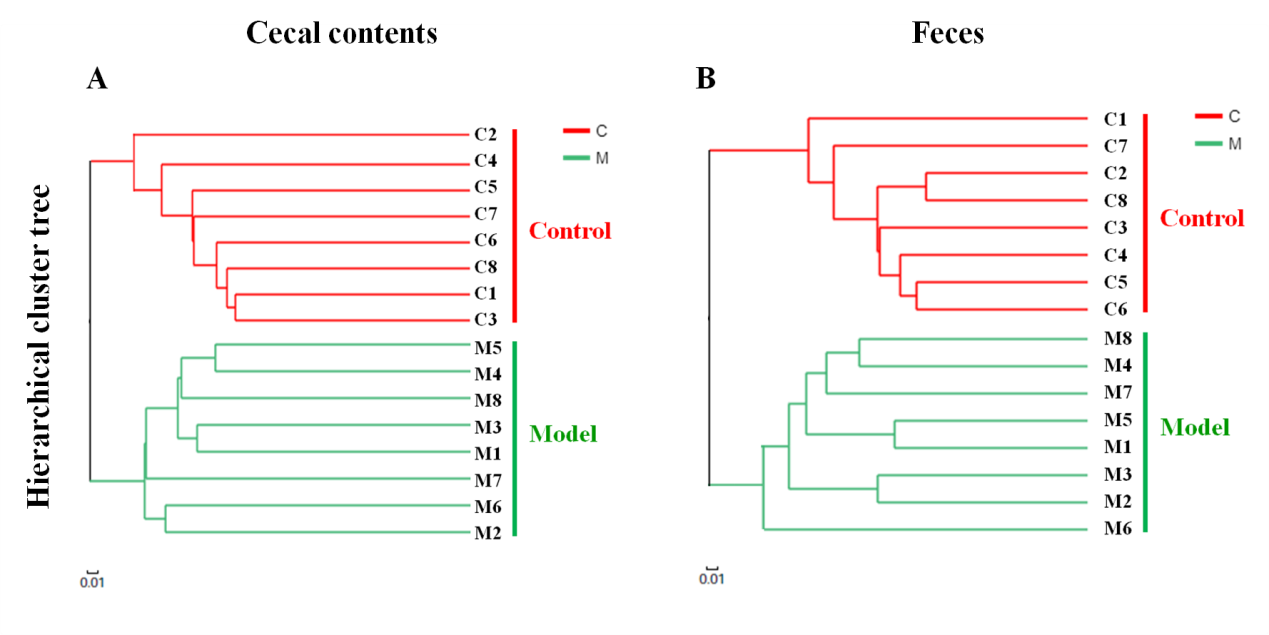


**Figure S3**. Hierarchical clustering analysis of cecal OTUs. The tree was established at a distance of 0.01. Red and green colors represent the control group and ISO-induced AMI group, respectively.


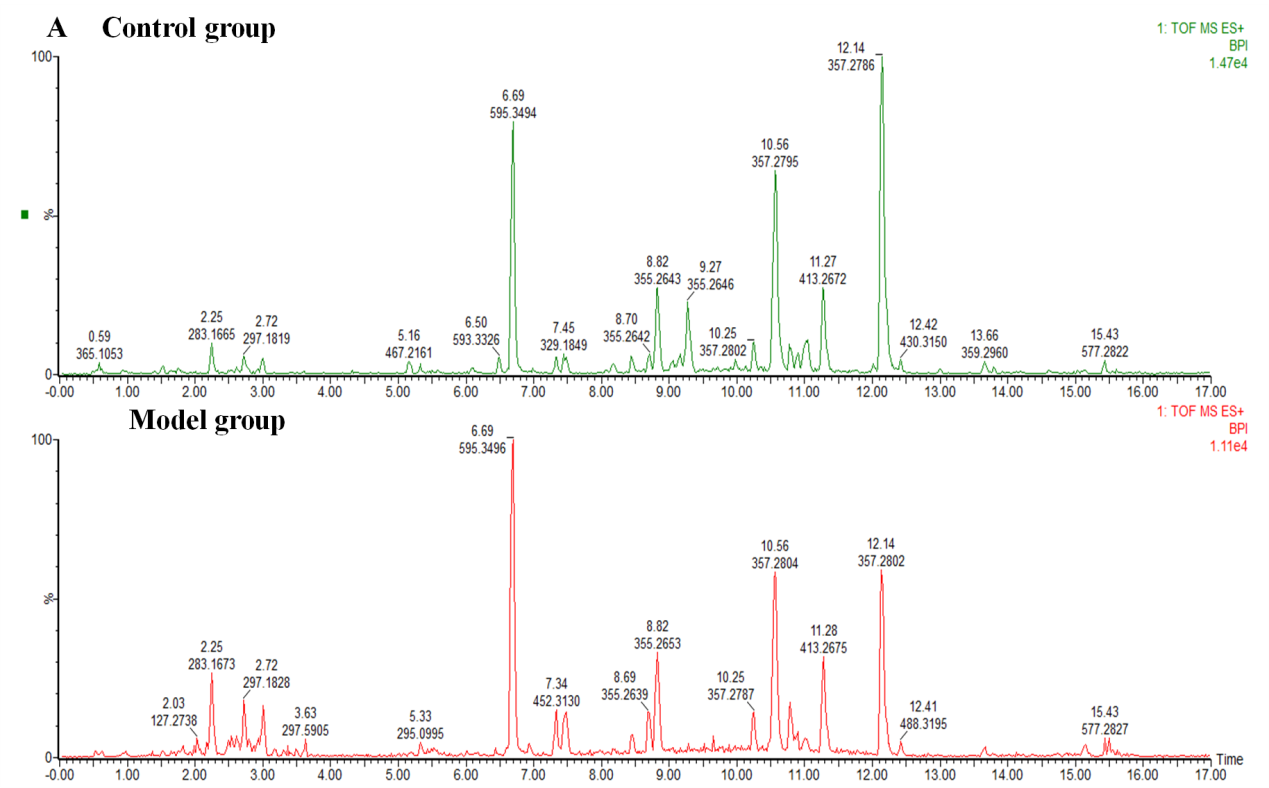


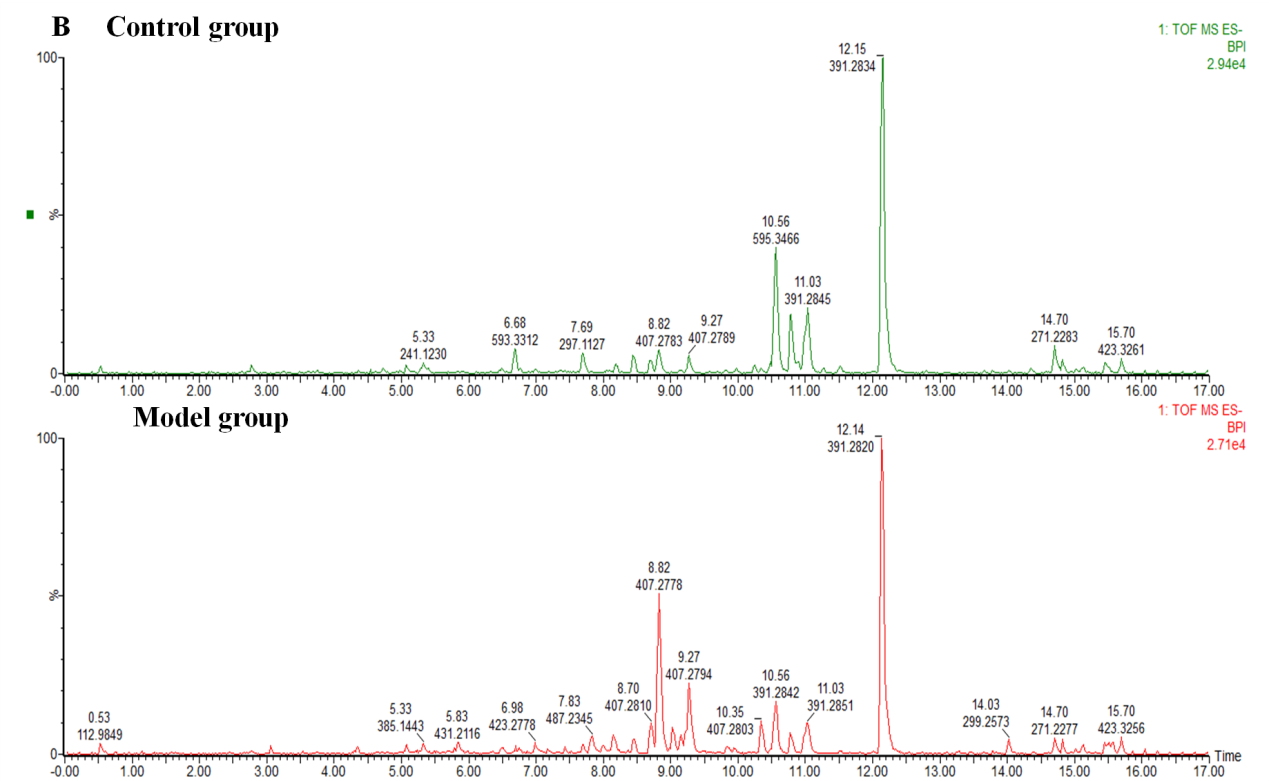


**Figure S4**. UPLC-QTOF-MS typical BPI chromatograms of cecal samples in (A) positive ion mode and (B) negative ion mode.


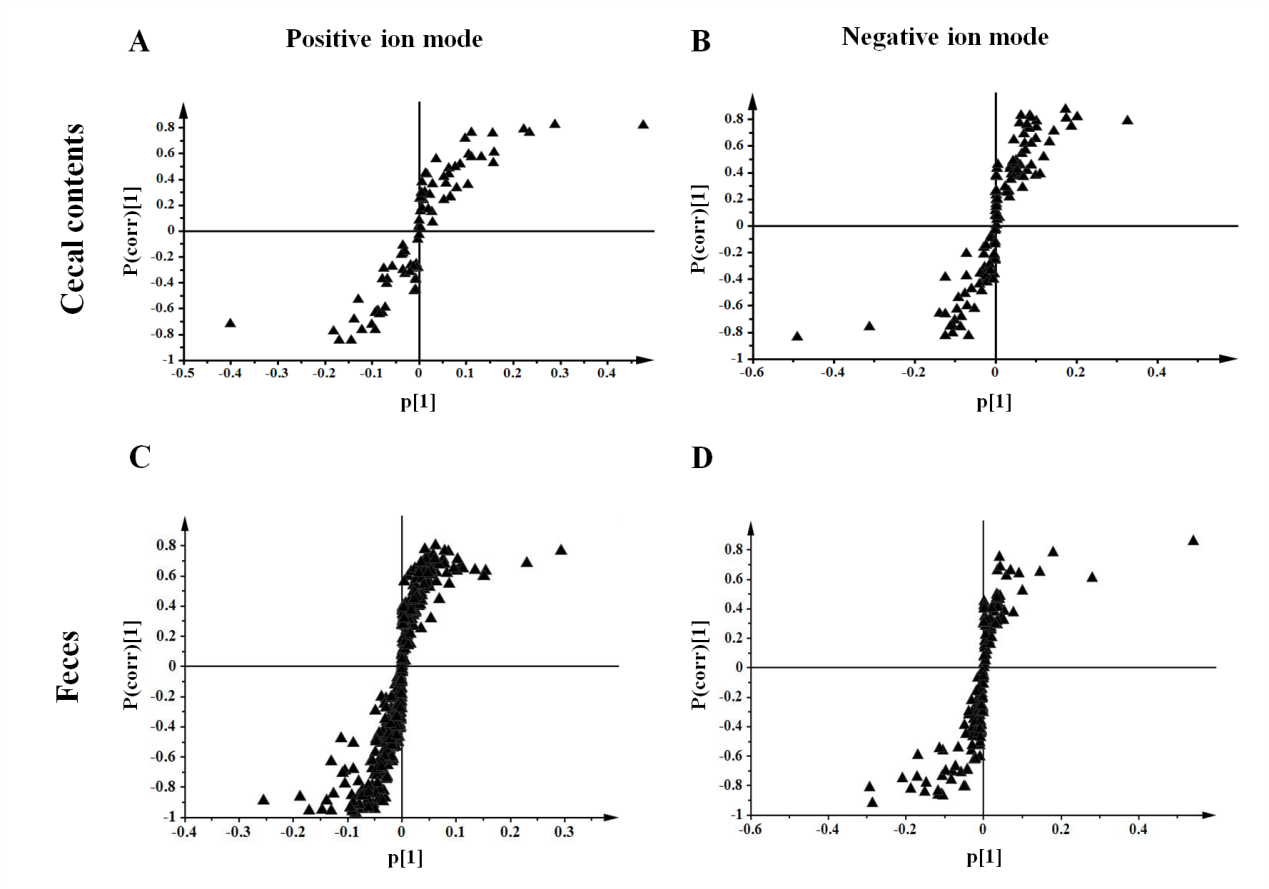


**Figure S5.** *S*-plot of cecal samples between the control and model group in (A) positive ion mode and (B) negative ion mode.


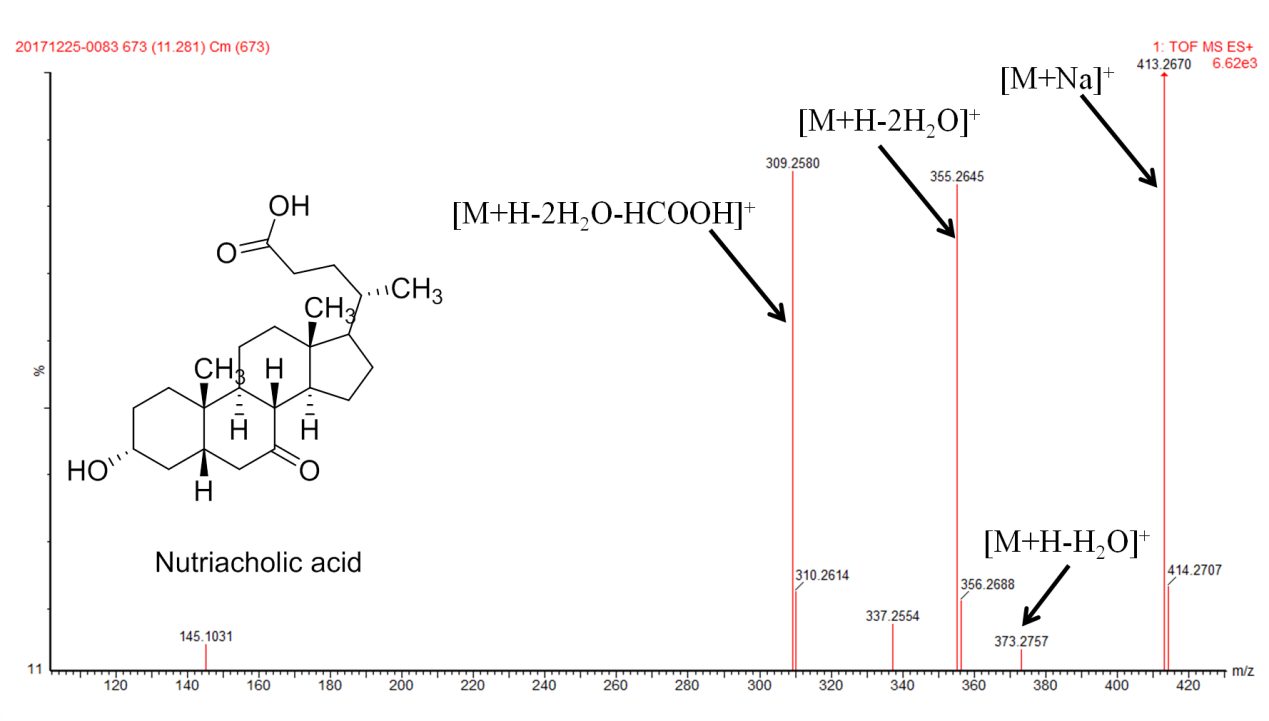


**Figure S6**. The MS^E^ spectra and possible fragmentation pathway of nutriacholic acid.


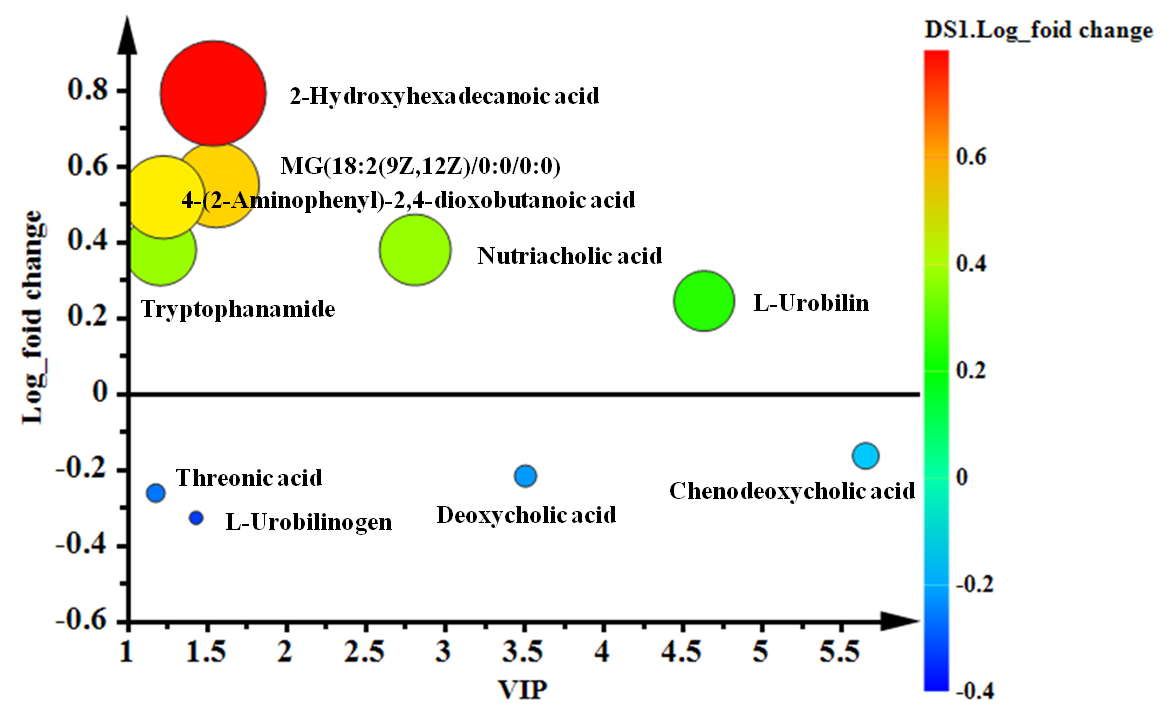


**Figure S7**. Fold changes and VIP of cecal biomarkers in the ISO-induced AMI group compared with the control group.


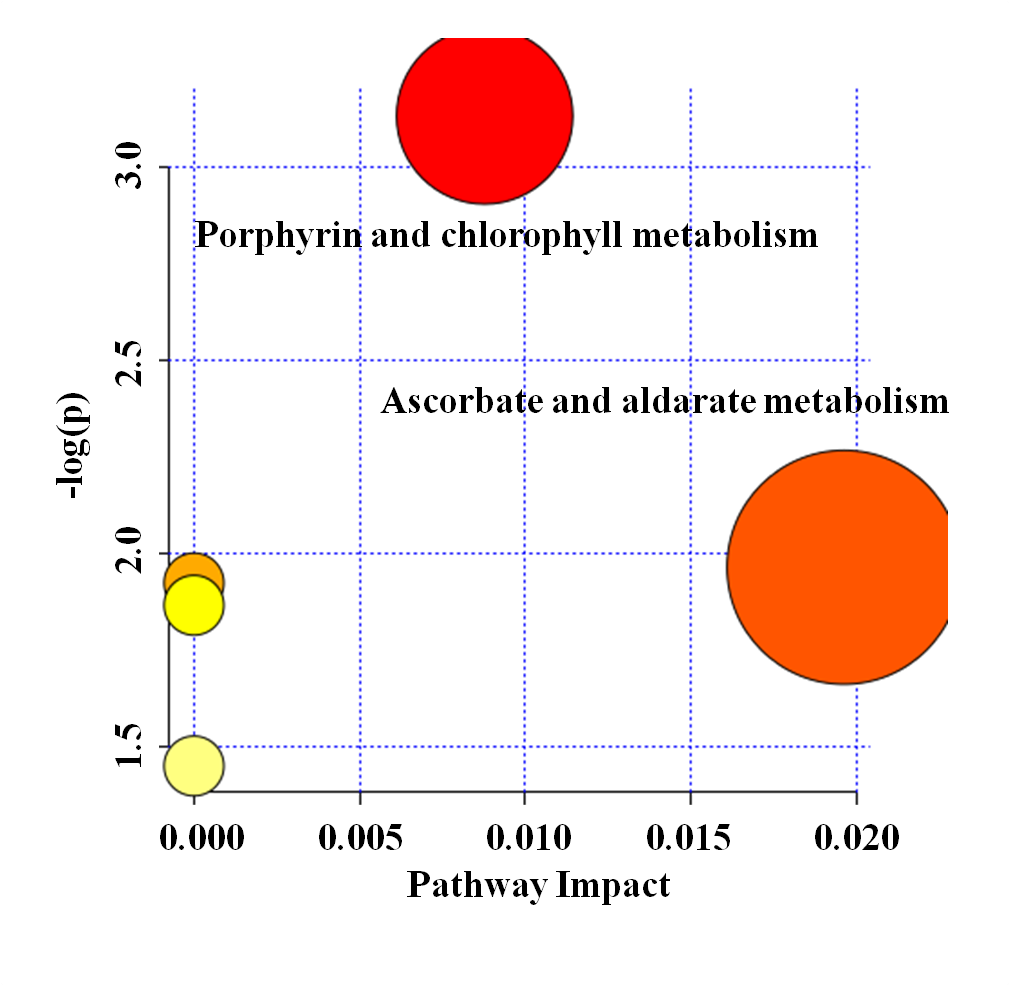


**Figure S8**. Summary of pathway analysis with MetPA.

## Supplementary Tables

**Table S1.** The information of antibodies for western blot analysis

| Antibodies | **Origin** | **No.** | **Company** | **Dilution ratio** |
| --- | --- | --- | --- | --- |
| IL-1β | Rabbit | ab9722 | Abcam | 1:1000 |
| TNF-α | Rabbit | ab6671 | Abcam | 1:1000 |
| Cleaved caspase 3 | Mouse | YM3431 | Immunoway | 1:1000 |
| Caspase 7 | Rabbit | 12827 | CST | 1:2000 |

**Table S2.** Fold changes and p-values of significant altered genera in cecal contents in ISO-induced AMI compared with control group

| **Altered fecal microbiota** | **Percent of**  **control group** | **Percent of**  **Model group** | **Fold**  **change** | ***p*-value** |
| --- | --- | --- | --- | --- |
| ***Ruminococcaceae UCG-005*** | 0.1335 | 0.0607 | 0.4546 | 0.00153 |
| ***Bacteroidales S24-7 group norank*** | 0.0549 | 0.1302 | 2.3738 | 0.00013 |
| ***Prevotellaceae UCG-003*** | 0.0040 | 0.0563 | 14.0153 | 0.00458 |
| ***Lachnospiraceae NK4A136 group*** | 0.0378 | 0.0080 | 0.2123 | 0.02351 |
| ***Ruminiclostridium 9*** | 0.0258 | 0.0154 | 0.5972 | 0.02172 |
| ***Prevotella 9*** | 0.0113 | 0.0282 | 2.4937 | 0.02729 |
| ***Ruminococcaceae UCG-014*** | 0.0274 | 0.0083 | 0.3023 | 0.00157 |
| ***Ruminococcus 1*** | 0.0064 | 0.0247 | 3.8461 | 0.00373 |
| ***Oscillibacter*** | 0.0196 | 0.0099 | 0.5023 | 0.04208 |
| ***Treponema 2*** | 0.0012 | 0.0260 | 21.2741 | 0.00007 |
| ***Rikenellaceae RC9 gut group*** | 0.0011 | 0.0144 | 12.8819 | 0.01648 |
| ***Lachnoclostridium*** | 0.0120 | 0.0030 | 0.2453 | 0.03273 |
| ***Bacteroides*** | 0.0013 | 0.0136 | 10.1879 | 0.00008 |

**Table S3**. The potential cecal biomarkers and their variation tendencies in ISO-induced AMI rats detected by UPLC-Q/TOF MS

| **NO.** | **Metabolites** | **RT (min)** | **m/z** | **Adduct Ion** | **M/C** | **Formula** | **Pathways** |
| --- | --- | --- | --- | --- | --- | --- | --- |
| **C1** | Threonic acid | 0.92 | 137.0510 | [M+ H ]^+^ | ↓^**^ | C_4_H_8_O_5_ | Ascorbate and aldarate metabolism |
| **C2** | Tryptophanamide | 2.06 | 226.1090 | [M+ Na ]^+^ | ↑^**^ | C_11_H_13_N_3_O | Tryptophan metabolism |
| **C3** | Unidentified | 2.76 | 162.0561 | [M+H]^+^ | ↑^**^ | C_9_H_7_NO_2_ | Unknown |
| **C4** | L-Urobilin | 6.71 | 595.3490 | [M+H]^+^ | ↑^***^ | C_33_H_46_N_4_O_6_ | Porphyrin and chlorophyll metabolism |
| **C5** | MG(18:2(9Z,12Z)/0:0/0:0) | 7.47 | 355.2677 | [M+H]^+^ | ↑^***^ | C_21_H_38_O_4_ | Fatty acid metabolism |
| **C6** | L-Urobilinogen | 10.56 | 619.3475 | [M+Na]^+^ | ↓^**^ | C_24_H_36_O_2_ | Porphyrin and chlorophyll metabolism |
| **C7** | Nutriacholic acid | 11.28 | 413.2670 | [M+Na]^+^ | ↑^***^ | C_24_H_38_O_4_ | Bile acid biosynthesis |
| **C8** | Chenodeoxycholic acid | 12.14 | 415.2830 | [M+Na]^+^ | ↓^***^ | C_24_H_40_O_4_ | Bile acid biosynthesis |
| **C9** | 4-(2-Aminophenyl)-2,4-dioxobutanoic acid | 2.79 | 206.0468 | [M-H] ^-^ | ↑^**^ | C_10_H_9_NO_4_ | Tryptophan metabolism |
| **C10** | Unidentified | 5.86 | 431.2094 | [M-H] ^-^ | ↑^***^ | - | Unknown |
| **C11** | Unidentified | 8.81 | 407.2785 | [M-H] ^-^ | ↑^***^ | - | Unknown |
| **C12** | Deoxycholic acid | 11.03 | 391.2836 | [M-H] ^-^ | ↓^***^ | C_24_H_40_O_4_ | Bile acid biosynthesis |
| **C8** | Chenodeoxycholic acid | 12.14 | 391.2829 | [M-H] ^-^ | ↓^***^ | C_24_H_40_O_4_ | Bile acid biosynthesis |
| **C13** | 2-Hydroxyhexadecanoic acid | 14.71 | 271.2273 | [M-H] ^-^ | ↑^***^ | C_16_H_32_O_3_ | Fatty acid metabolism |

M/C: the model group compared to the control group; ↑: up-regulated, ↓: down-regulated. ^**^ *p* < 0.01, ^***^ *p* < 0.001.

**Table S4:** Pearson’s correlation matrix between altered cecal microbiota/perturbed cecal metabolites and over-expression proteins between the control and model groups.

| **Altered gut microbiota** | **TNF-α** | **IL-1β** | **Cleaved caspase-3** | **caspase-7** |
| --- | --- | --- | --- | --- |
| ***Ruminococcaceae UCG-005*** | 0.32423 | 0.37258 | 0.58812 | -0.32370 |
| ***Bacteroidales S24-7 group norank*** | 0.64001 | 0.51036 | 0.24655 | **0.88133**^*^ |
| ***Prevotellaceae UCG-003*** | 0.42347 | 0.16602 | 0.06421 | 0.39094 |
| ***Lachnospiraceae NK4A136 group*** | -0.12188 | -0.43740 | 0.37516 | 0.11044 |
| ***Ruminiclostridium 9*** | 0.09441 | 0.37112 | -0.54652 | 0.70137 |
| ***Prevotella 9*** | 0.09951 | -0.01186 | 0.51655 | 0.41433 |
| ***Ruminococcaceae UCG-014*** | -0.47813 | -0.42034 | -0.02996 | 0.10709 |
| ***Ruminococcus 1*** | 0.33364 | 0.12676 | 0.46030 | 0.68787 |
| ***Oscillibacter*** | 0.23627 4 | 0.18582 | 0.45443 | 0.64610 |
| ***Treponema 2*** | 0.71493 | 0.87761 | 0.33347 | 0.22265 |
| ***Rikenellaceae RC9 gut group*** | -0.08438 | 0.12904 | -0.22093 | 0.69919 |
| ***Lachnoclostridium*** | -0.22769 | -0.60307 | 0.30683 | -0.18045 |
| ***Bacteroides*** | **0.89094**^*^ | **0.88589**^*^ | 0.30823 | 0.73228 |
| **Cecal biomarkers** | **TNF-α** | **IL-1β** | **Cleaved caspase-3** | **caspase-7** |
| **C1** | 0.06119 | -0.11700 | -0.32746 | 0.11905 |
| **C2** | -0.47691 | -0.32794 | -0.19465 | 0.23110 |
| **C4** | 0.07085 | -0.25827 | -0.03242 | 0.06680 |
| **C5** | -0.44226 | -0.67826 | -0.29831 | -0.65558 |
| **C6** | -0.68422 | -0.58311 | **-0.88540**^*^ | -0.47880 |
| **C7** | -0.90402 | -0.81679 | -0.60345 | -0.85653 |
| **C8** | -0.26972 | 0.02182 | -0.87608 | 0.18336 |
| **C9** | -0.96223 | -0.94728 | -0.63429 | -0.46807 |
| **C12** | 0.14028 | 0.20245 | 0.58364 | -0.21486 |
| **C13** | 0.18044 | -0.11334 | -0.05081 | 0.08023 |

Red suggests specific gut microbiota/cecal metabolites were highly correlated with over-expressed proteins. **P* < 0.05
